# Down-regulation of SOCS6: an unfavorable prognostic factor for gastrointestinal stromal tumor proven by survival analysis

**DOI:** 10.1186/s13000-021-01172-6

**Published:** 2021-12-12

**Authors:** Jun Ouyang, Tailai An, Yan Wang, Xiaofang Lu, Yawei Zhang, Xiaokun Wang, Xinhua Zhang, Changhua Zhang

**Affiliations:** 1 Center of Digestive Diseases, The Seventh Affiliated Hospital, Sun Yat-sen University, Zhenyuan Road 628, Guangming District, Shenzhen, Guangdong China; 2 Department of Gastrointestinal Surgery, The First Affiliated Hospital, Sun Yat-sen University, Zhongshan Road 58, Yuexiu District, Guangzhou, Guangdong China; 3 Department of Hepatobiliary and Pancreatic Surgery, Shenzhen People’s Hospital, Guangdong Shenzhen, China; 4 Department of Radiology, Shenzhen People’s Hospital, Shenzhen, Guangdong China; 5 Department of Pathology, The Seventh Affiliated Hospital, Sun Yat-sen University, Shenzhen, Guangdong China

**Keywords:** Gastrointestinal stromal tumor, SOCS6, Overall survival, Recurrence-free survival, Cox proportional regression model analysis

## Abstract

**Background:**

Many studies reporting that down-regulation of SOCS6 plays vital roles in promoting progression of malignant tumors have been published. The present study was performed to evaluate whether SOCS6 was significantly associated with prognosis of GIST patients.

**Methods:**

Immunohistochemical staining was accomplished to evaluate the expression levels of SOCS6 among GIST patients. The impacts of SOCS6 expression on overall survival (OS) and recurrence-free survival (RFS) of GIST patients were assessed by Cox proportional hazard regression model analysis and Kaplan-Meier curve analysis.

**Results:**

It was demonstrated that the expression level of SOCS6 was significantly associated with tumor size (*P*=0.001). Then according to Kaplan-Meier curve analysis, low expression of SOCS6 was significantly correlated with worse OS and RFS of GIST patients. Ultimately, it was revealed by Cox proportional regression model analysis that low expression of SOCS6 was an independent predictive factor for OS and RFS.

**Conclusions:**

Low expression of SOCS6 was an independent prognostic factor for GIST, suggesting its potential as a novel biomarker predicting survival of GIST patients.

## Background

As the most common stromal tumor originating from the gastrointestinal tract, gastrointestinal stromal tumor (GIST) accounts for 0.1-3% of all gastrointestinal malignant tumors and 6% of the sarcomas [1]. Globally, the annual incidence of GIST is 10/1,000,000 [2]. It has been revealed by genomic sequencing that activated mutations of receptor protein tyrosine kinase (RPTKs) or platelet-derived growth factor receptor-α(PDGFRA) occur in approximately 85-90% of GISTs [3]. Although the application of receptor kinase KIT and PDGFRA inhibitor could efficiently control the progression of 80-90% GISTs, about 50% of GIST patients experience secondary drug resistance within 2 years [4, 5]. As far as we know, curative surgery remains the primary treatment for resectable GISTs despite the fact more than 50% of patients with advanced GISTs will experience tumor recurrences [2]. Thus, seeking more biomarkers associated with prognosis and clinicopathological features of GISTs is still a meaningful work for us.

Abnormally persistent activation of growth factor receptor signaling pathways has been reported to participate in a series of pathological processes such as autoimmune diseases and malignant tumors. Negative feedback regulation plays vital roles in maintaining the balance between pro-proliferative signals and anti-proliferative signals. Persistent pro-proliferative signals triggered by loss of function (LOF) of the negative feedback regulation mechanisms would lead to excessive proliferation of cells and even occurrence of malignant tumors. As one of the ubiquitous E3 ubiquitin ligases, suppressor of cytokine signaling 6 (SOCS6) could promote the ubiquitin-mediated degradation of proteins by binding with phosphorylated tyrosine receptors or signaling proteins [6, 7]. The locus (18q22.2) where human SOCS6 resides is commonly associated with malignant tumors [8]. Deletion of the genes located at 18q22.2 has been reported to occur in multiple malignant tumors such as lung spuamous cell carcinoma, hepatocellular carcinoma, prostate cancer and leukemia and this deletion is significantly associated with poor prognosis [9–12]. However, it remains unclear whether SOCS6 affects survival of GIST patients. Thus, we performed the present study to assess the expression of SOCS6 in GIST tissues and evaluate the capability of SOCS6 expression to predict prognosis of GIST patients.

## Materials and methods

### Patients and clinical samples

GIST patients having undergone curative surgery at the Department of Gastrointestinal Surgery, The First Affiliated Hospital, Sun Yat-sen University between January 2000 and December 2014 were retrospectively reviewed. The diagnosis of GIST was made according to the Chinese and NCCN guidelines on GIST. According to the Chinese and NCCN guidelines on GIST, morphological conformity and results of immunohistochemical staining (CD117, CD34 and DOG1 positivity) are the basis of diagnosing GISTs, and for patients with rare types, both mutations of Kit and PDGFRAT and expression of SDHB were detected to confirm the diagnosis of GIST. Two senior pathologists independently confirmed the diagnosis. The inclusion criteria of this study were as follows: curative resection; no preoperative distant metastasis; no preoperative or postoperative application of TKI; without other malignant tumors; complete clinicopathological data. The following information of each included patient was retrieved from his or he medical records: gender, age, tumor size, tumor location, necrosis of tumor, mitotic index (per 50 high power fields under old microscope or 21 high power fields under new microscope; equal to 5 square milimeters). Tumor risk grade of each patient was assessed according to the modified National Institutes of Health (NIH) consensus [13]. Declaration of Helsinki was adhered to during the whole process of this study. Informed consent in written form was obtained from each individual patient before the study.

### Immunohistochemical staining and scoring

**Fig. 1:**
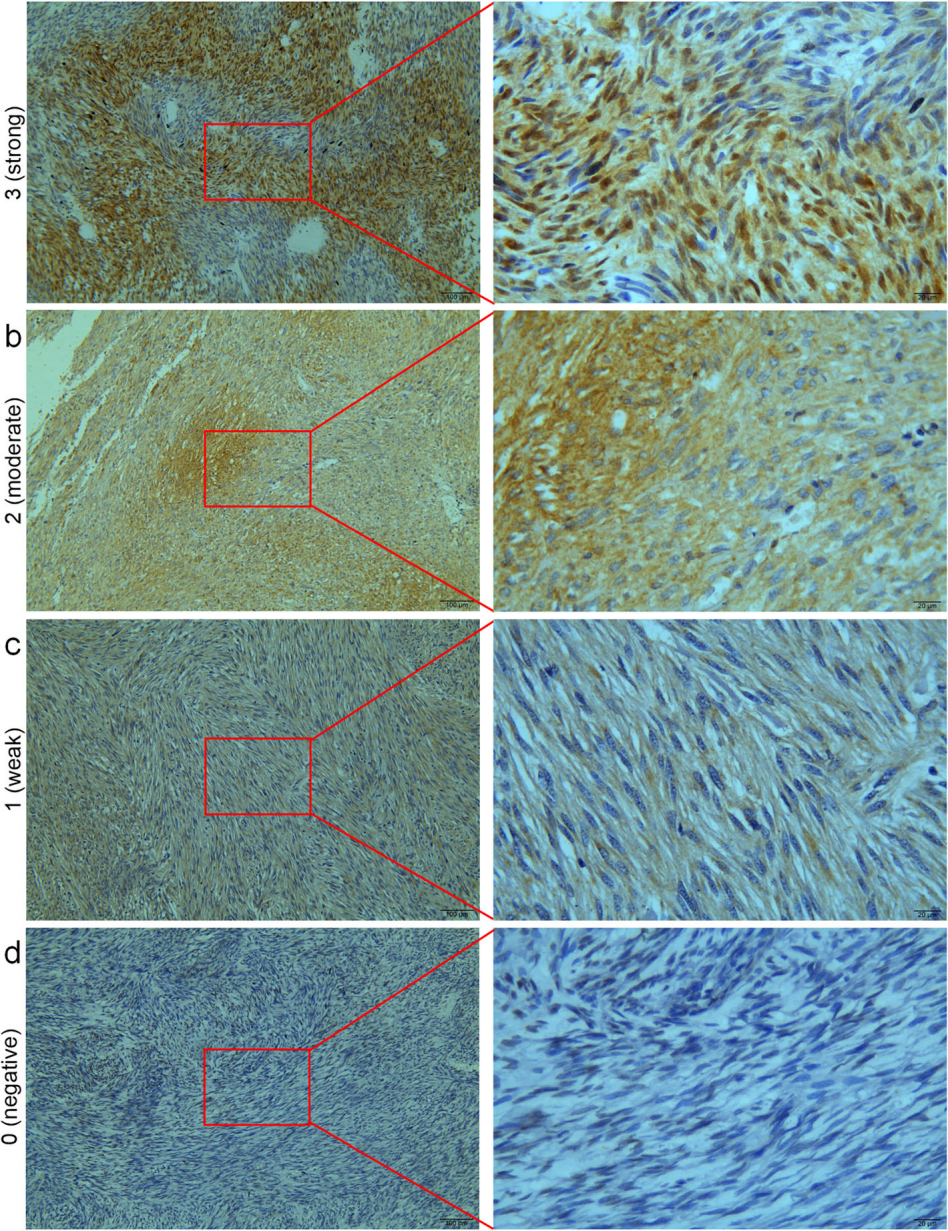
Different staining intensities of SOCS6 in GIST tissues identified by immunohistochemical staining. **A** Strong staining intensity. **B** Moderate staining intensity. **C** Weak staining intensity. **D** Negative staining intensity

The expression level of SOCS6 in GIST tissue was evaluated by immunohistochemical staining. Slides bearing GIST tissues were initially embedded in paraffin. GIST tissues were first deparaffined by xylene and then rehydrated using alcohol of different concentrations (100%, 95%, 85% and 75%). Then after rehydration, slides were soaked in 0.3% hydrogen peroxide for 20 min to block endogenous peroxidase activity. And then GIST tissues were blocked by 10% bovine serum albumin (BSA) for 30 min. Subsequently, GIST tissues were incubated with SOCS6-specific antibody (1:100, ab197335, Abcam, Cambridge, MA, USA) at 4℃ overnight. On the second day, GIST tissues were incubated with the biotinylated secondary antibody (Biotin-conjugated Affinipure Goat Anti-Rabbit IgG (H+L), SA00004-2, Proteintech, Wuhan, China) for 30 min. Subsequently, 3,3,-diaminobenzidine (DAB) (GK600510, Genomics Shanghai, China) was used as the chromogenic substrate to visualize the antibody-conjugated SOCS6. Ultimately, GIST tissues were counter stained using hematoxylin. The stained GIST tissues were semi-quantitatively scored independently by two pathologists without priorly knowing patients^,^ clinicopathological information. Staining intensities were classified as follows: strong staining (3, shown in Fig. 1A), moderate staining (2, shown in Fig. 1B), weak staining (1, shown in Fig. 1C) and negative staining (0, shown in Fig. 1D). While positive cell percentage scores were defined as follows: 0 (<5%), 1 (5-24%), 2 (25-50%) and 3 (>50%). Finally, the scores were obtained by multiplying the staining intensity score and positive cell percentage score. High expression was defined when the total score was ≥4 and <4 was defined as low expression.

### Statistical analysis

STATA14.0 software (Stata Corp LP, College Station, Texas) was used to accomplish relevant statistical analyses. Measurement data were compared by Mann-Whitney U test while categorical variables using χ2 test or Fisher^,^s exact test. Overall survival (OS) was defined as the duration between curative surgery and death no matter the cause while the time length between curative surgery and tumor recurrence was recorded as recurrence-free survival (RFS). Survival curves of GIST patients were compared by Kaplan-Meier curve analysis and further tested by log-rank test. Both univariate and multivariate Cox proportional hazard regression model analyses were accomplished to calculate hazard ratio (HR) and 95% confidential interval (95%CI) and to identify independent prognostic factors for GIST patients. All the tests accomplished in this study were two-sided in nature and a P value <0.05 was considered as statistically significant.

## Results

### Associations between SOCS6 expression and clinicopathological variables

**Fig. 2:**
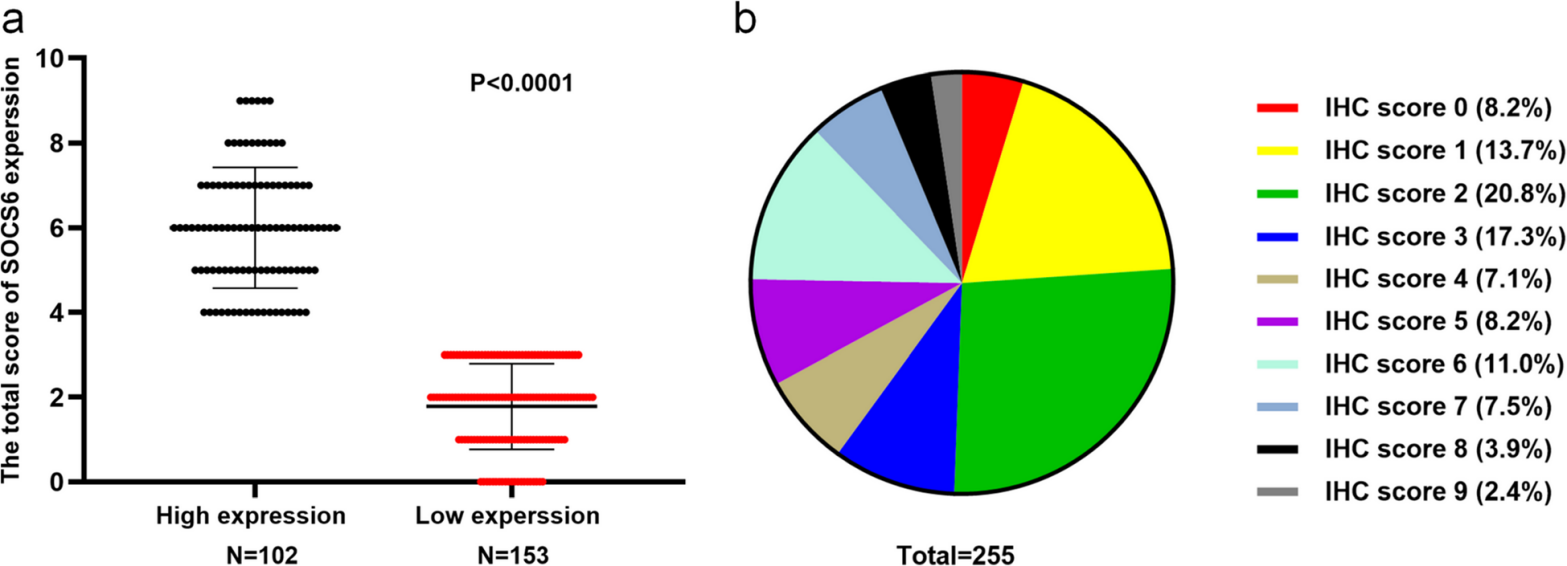
Distribution of SOCS6 IHC scores among GIST patients. **A** High expression vs. low expression of SOCS6 among GIST patients. **B** Distribution of different IHC scores among the included GIST patients

Though immunohistochemical staining, we could find that SOCS6 was mainly distributed in cytoplasm and nucleus. Of the 255 GIST patients, 102 ones were identified to have high SOCS6 expression (mean score: 6±1.43) and 153 ones with low SOCS6 expression (mean score: 1.78±1.01)(Fig. 2A and B). The associations between SOCS6 expression and clinicopathological variables were presented in Table 1, from which we could see that SOCS6 expression was significantly associated with tumor size (*P*=0.001). However, SOCS6 expression was not significantly correlated with other variables including age, gender, tumor location, necrosis of tumor, mitotic index and NIH risk grade.

**Table 1:** Associations between SOCS6 expression and clinicopathological variables

	High expression (*N*=102)	Low expression (*N*=153)	P
Median age (years)	56	56	0.28
Gender			0.44
Male	55(53.9%)	91(59.5%)	
Female	47(46.1%)	62(40.5%)	
Tumor location			0.48
Stomach	66(64.7%)	94(61.4%)	
Small intestine	31(33.3%)	51(33.3%)	
Colorectum	2(2.0%)	8(5.2%)	
Median tumor size(cm)	3.7	5.5	< 0.001
Necrosis of tumor			0.58
No	73(71.6%)	104(68.0%)	
Yes	29(28.4%)	49(32.0%)	
Mitotic index ( per 50 HPF)			0.48
<5	65(63.7%)	92(60.1%)	
5~10	20(19.6%)	26(17.0%)	
>10	17(16.7%)	35(22.9%)	
NIH risk grade			0.70
Extremely low	12(11.8%)	12(7.8%)	
Low	28(27.5%)	39(25.5%)	
Moderate	21(20.6%)	35(22.9%)	
High	41(40.2%)	67(43.8%)	
Morphology			0.57
Spindle	93(91.2%)	144(94.1%)	
Epithelioid	3(2.9%)	2(1.3%)	
Mixed	6(5.9%)	7(4.6%)	
CD117			0.74
Positive	99(97.1%)	147(96.1%)	
Negative	3(2.9%)	6(3.9%)	
CD34			1.00
Positive	89(87.3%)	134(87.6%)	
Negative	13(12.7%)	19(12.4%)	
DOG1			0.73
Positive	38(37.3%)	50(32.7%)	
Negative	2(2.0%)	4(2.6%)	
Unknown	62(60.8%)	99(64.7%)

### The impacts of SOCS6 on OS

**Fig. 3:**
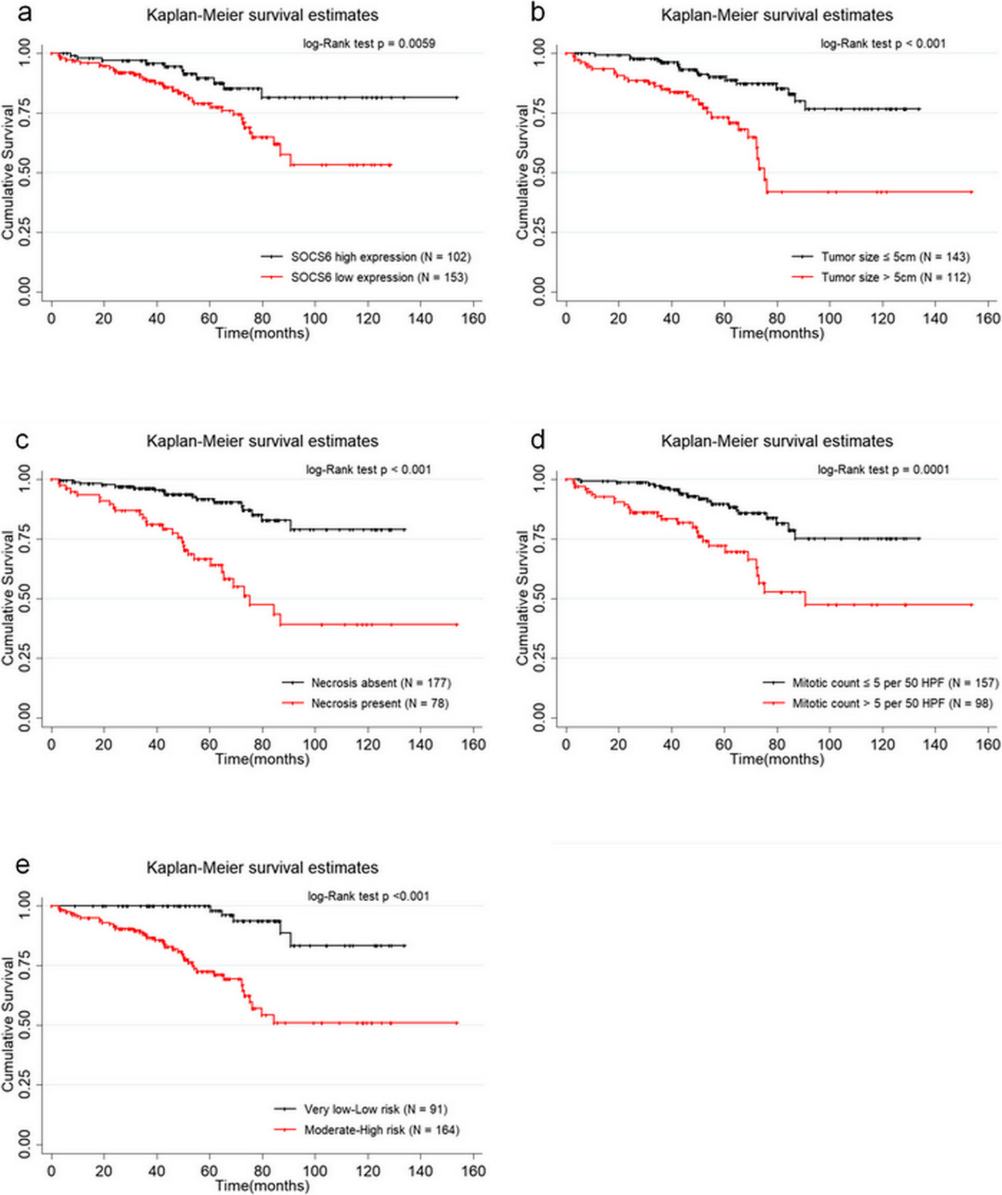
The impacts of SOCS expression, tumor size, necrosis of tumor, mitotic index, NIH risk grade on OS assessed according to Kaplan-Meier curve analysis and log-rank test. **A** Impact of SOCS6 expression on OS of GIST patients. **B** The impact of tumor size on OS of GIST patients. **C** The impact of necrosis of tumor on OS of GIST patients. **D** The impact of mitotic index on OS of GIST patients. **E** The impact of NIH risk grade on OS of GIST patients

Follow-ups of the 255 GIST patients range from 2 to 156 months and the 1-year, 3-year and 5-year OS of these 255 patients were 96.81%, 92% and 83.1% respectively. The mean survival time of these 255 patients was 119.82±4.38 months. For patients with high SOCS6 expression, the 1-year, 3-year and 5-year OS were 97.79%, 95.75% and 89.62% respectively and the mean survival was 135.00±5.30 months. Whereas, for patients with low SOCS6 expression, the 1-year, 3-year and 5-year OS were 96.04%, 89.49% and 78.95% respectively and the mean survival was 95.56±4.56 months. According to Kaplan-Meier curve analysis, low SOCS6 expression (*P*=0.0059), tumor size>5 cm (*P*<0.001), necrosis of tumor (*P*<0.001), Mitotic index>5/50HPF(*P*=0.0001), moderate or high NIH risk grade (*P*<0.001) were significantly associated with worse OS of GIST patients (shown in Fig. 3A and E). Then it was revealed by univariate Cox proportional hazard regression model analysis that tumor size (*P*<0.001, HR=3.67, 95%CI: 1.99~6.74), necorsis of tumor (*P*<0.001, HR=4.50, 95%CI:2.48~8.18), mitotic index (*P*<0.001, HR=3.13, 95%CI: 1.75~5.62), moderate or high NIH risk grade (*P*<0.001, HR=7.37, 95%CI: 2.90~18.73) and low SOCS6 expression (*P*=0.008, HR=2.51, 95%CI: 1.27~4.93) were significantly associated with OS of GIST patients (shown in Table 2). Subsequently, tumor size (*P*=0.007, HR=2.49, 95%CI: 1.27~4.56), necorsis of tumor (*P*=0.001, HR=3.03, 95%CI: 1.54~5.95), mitotic index (*P*=0.023, HR=2.11, 95%CI: 1.11~4.02), and low SOCS6 expression (*P*=0.009, HR=2.58, 95%CI: 1.27~5.24) were demonstrated by multivariate Cox proportional hazard regression model analysis to be independent predictive factors for OS (shown in Table 2).

**Table 2:** Cox proportional-hazard regression model analysis for overall survival

			Univariate		Multivariate	
	3-year OS	5-year OS	HR(95%CI)	P	HR(95%CI)	P
Age(years)						
≤65	92.43%	83.68%	reference		reference	
>65	90.58%	81.88%	1.32(0.70~2.46)	0.390	1.54(0.81~2.93)	0.187
Gender						
Male	89.77%	80.46%	reference		reference	
Female	95.01%	86.78%	0.64(0.35~1.16)	0.141	0.61(0.32~1.12)	0.115
Tumor location						
Stomach	91.14%	86.00%	reference		reference	
Non-stomach	93.43%	78.49%	1.75(0.98~3.11)	0.058	1.20(0.66~2.20)	0.550
Tumor size(cm)						
≤5	96.17%	90.07%	reference		reference	
>5	86.41%	73.38%	3.67(1.99~6.74)	<0.001	2.40(1.27~4.56)	0.007
Necrosis of tumor						
No	96.39%	91.67%	reference		reference	
Yes	82.47%	66.62%	4.50(2.48~8.18)	<0.001	3.03(1.54~5.95)	0.001
Mitotic index (per 50 HPF)						
≤5	96.47%	89.78%	reference		reference	
>5	84.74%	72.18%	3.13(1.75~5.62)	<0.001	2.11(1.11~4.02)	0.023
NIH risk grade						
Extremely low or low	100%	100%	reference			
Moderate or high	87.41%	72.60%	7.37(2.90~18.73)	<0.001		
SOCS6 expression						
High	95.75%	89.62%	reference		reference	
Low	89.49%	78.95%	2.51(1.27~4.93)	0.008	2.58(1.27~5.24)	0.009

### The impacts of SOCS6 expression on RFS

**Fig. 4:**
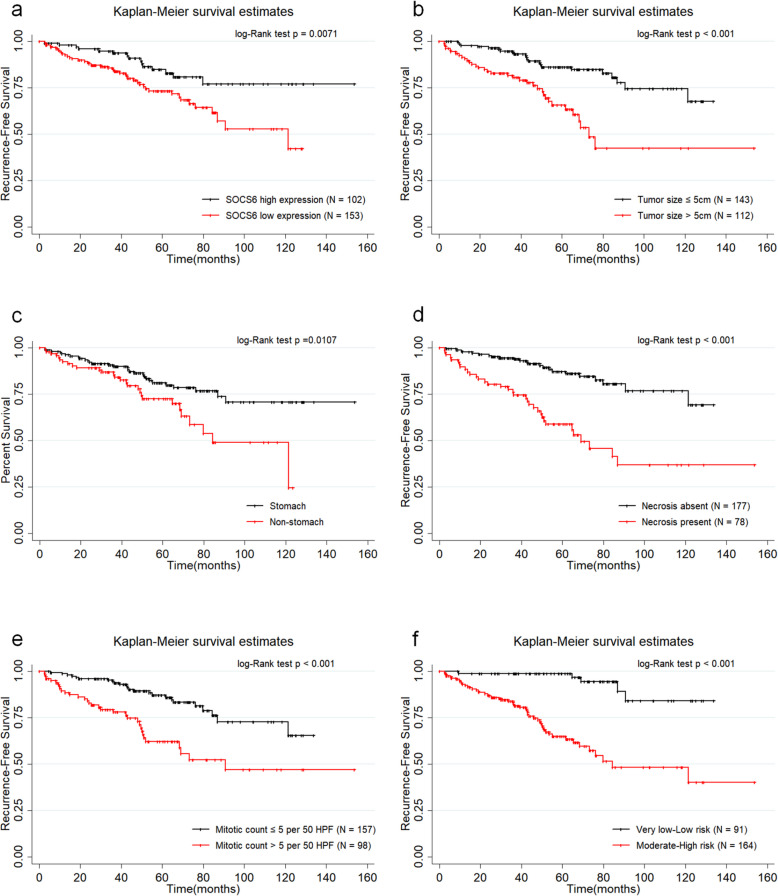
The impacts of SOCS expression, tumor size, necrosis of tumor, mitotic index, NIH risk grade on RFS assessed according to Kaplan-Meier curve analysis and log-rank test. **A** Impact of SOCS6 expression on RFS of GIST patients. **B** The impact of tumor size on RFS of GIST patients. **C** The impact of tumor location on RFS of GIST patients. **D** The impact of necrosis of tumor on RFS of GIST patients. **E** The impact of mitotic index on RFS of GIST patients. **F** The impact of NIH risk grade on RFS of GIST patients

The 1-year, 3-year and 5-year RFS of these 255 patients were 95.21%, 89.30% and 77.98% respectively. The median RFS of the 255 patients was 114.02±4.61 months. For patients with high SOCS6 expression, the 1-year, 3-year and 5-year RFS were 96.96%, 94.81% and 84.79% respectively and the mean RFS was 129.49±5.73 months. Whereas, for patients with low SOCS6 expression, the 1-year, 3-year and 5-year RFS were 93.36%, 85.62% and 73.30% respectively and the mean RFS was 91.27±4.64 months. According to Kaplan-Meier curve analysis, low SOCS6 expression (*P*=0.0071), non-stomach GIST (*P*=0.0107), tumor size>5 cm (*P*<0.001), necrosis of tumor (*P*<0.001), Mitotic index>5/50HPF(*P*<0.001), moderate or high NIH risk grade (*P*<0.001) were significantly associated with worse RFS of GIST patients (shown in Fig. 4A and F). Then it was revealed by univariate Cox proportional hazard regression model analysis that tumor size (*P*<0.001, HR=3.18, 95%CI: 1.84~5.50), tumor location (*P*=0.012, HR=1.95, 95%CI: 1.16~3.30), necorsis of tumor (*P*<0.001, HR=3.93, 95%CI:2.31~6.68), mitotic index (*P*<0.001, HR=2.93, 95%CI: 1.73~4.97), moderate or high NIH risk grade (*P*<0.001, HR=8.89, 95%CI: 3.53~22.36) and low SOCS6 expression (*P*=0.009, HR=2.20, 95%CI: 1.22~3.98) were significantly associated with RFS of GIST patients (shown in Table 3). Subsequently, tumor size (*P*=0.014, HR=2.06, 95%CI: 1.16~3.68), necorsis of tumor (*P*=0.002, HR=2.60, 95%CI: 1.44~4.71), mitotic index (*P*=0.020, HR=2.60, 95%CI: 1.44~4.71), and low SOCS6 expression (*P*=0.018, HR=2.10, 95%CI: 1.14~3.89) were demonstrated by multivariate Cox proportional hazard regression model analysis to be independent predictive factors for RFS (shown in Table 3).

**Table 3:** Cox proportional-hazard regression model analysis for for recurrence-free survival

			Univariate		Multivariate	
	3-year RFS	5-year RFS	HR(95%CI)	P	HR(95%CI)	P
Age (years)						
≤65	89.50%	78.77%	reference		reference	
>65	88.72%	75.62%	1.25(0.70~2.23)	0.452	1.41(0.78~2.53)	0.255
Gender						
Male	87.8%	75.60%	reference		reference	
Female	91.32%	80.87%	0.69(0.40~1.18)	0.181	0.72(0.41~1.25)	0.243
Tumor location						
Stomach	90.78%	81.16%	reference		reference	
Non-stomach	86.79%	72.53%	1.95(1.16~3.30)	0.012	1.46(0.85~2.52)	0.171
Tumor size(cm)						
≤5	94.86%	86.26%	reference		reference	
>5	81.77%	65.76%	3.18(1.84~5.50)	<0.001	2.06(1.16~3.68)	0.014
Necrosis of tumor						
No	94.63%	87.28%	reference		reference	
Yes	77.49%	58.83%	3.93(2.31~6.68)	<0.001	2.60(1.44~4.71)	0.002
Mitotic index (per 50 HPF)						
≤5	95.35%	87.26%	reference		reference	
>5	79.41%	62.15%	2.93(1.73~4.97)	<0.001	1.99(1.11~3.55)	0.020
NIH risk grade						
Extremely low or low	98.89%	98.89%	reference			
Moderate or high	83.85%	64.90%	8.89(3.53~22.36)	<0.001		
SOCS6 expression						
High	94.81%	84.79%	reference		reference	
Low	85.62%	73.30%	2.20(1.22~3.98)	0.009	2.10(1.14~3.89)	0.018

## Discussion

As a member of cytokine signaling inhibitor protein family, SOCS6 is characterized by a functional Src homologous domain (SH2), a SOCS box located at the C-terminus, and a binding region of various length and sequence at the N-terminus [8]. The SH2 domain is responsible for regulating cell signaling pathways via participating in the interaction between signaling proteins and phosphorylated tyrosine residues while the SOCS box serves as an elonginB/C-independent binding domain that links SOCS proteins to E3 ubiquitin ligases and proteasomes [8]. Currently, SOCS6 is considered as a specific regulator of receptor tyrosine kinase signaling pathway. In Ba/F3, MEF and COS-7 cells, overexpression of SOCS6 could inhibit cell proliferation through inhibiting expression of KIT and phosphorylation of ERK1/2 and p38 but does not affect the phosphorylation of AKT and STAT5 [14]. Thus, SOCS6 accomplishes its regulatory roles not only through ubiquitin-mediated degradation of receptor tyrosine kinases but also via negatively regulating downstream signaling proteins of receptor tyrosine kinase such as ERK1/2 and p38.

By far SOCS6 has been reported to be deleted in many malignant tumors. The absence of SOCS6 in primary lung squamous carcinoma was reported to be significantly associated with worse survival of patients [9]. Yuan et al. reported that expression of SOCS6 in prostate cancer was down-regulated and its low expression in prostate cancer was significantly associated with advanced stage and lymph node metastasis [11]. Furthermore, Yuan et al. had also proven that low SOCS6 expression was an independent prognostic factor for prostate cancer [11]. Similarly, SOCS6 was down-regulated in hepatocellular carcinoma and low expression of SOCS6 was significantly associated with progression, high recurrence risk and worse recurrence-free survival of hepatocellular carcinoma [10]. Besides its roles in lung squamous carcinoma, prostate cancer and hepatocellular carcinoma, SOCS6 could also inhibit the growth of gastric cancer, non-small cell lung cancer and cervical cancer via inhibiting angiogenesis, suppressing tumor cell proliferation and promoting apoptosis [15]. Furthermore, SOCS6 had been reported to regulate sensitivity of cancer cells to radiotherapy and chemotherapy [15–17]. And the epigenetic modification of the promoter region such as methylation has been proven to lead to down-regulation or loss of SOCS6 expression [18]. A more recent study reported that miR-k12-1-5p could lead to decreased expression of SOCS6 in Kaposi’s sarcoma [19]. In glioblastoma, up-regulation of miR-494 could result in reduced expression of SOCS6 [20]. While in bladder cancer cells, lncRNA NBAT1 could regulate SOCS6 expression via miR-21-5p [21]. Thus, considering all these aforementioned studies, we could draw the conclusion that SOCS6 could act as a tumor suppressor gene in many kinds of cancers and the expression of SOCS6 was regulated by methylation of its promoter region and was directly or indirectly controlled by miRNA and lncRNA.

In the present study, it was revealed that SOCS6 expression in GIST was significantly associated with tumor size and was an independent prognostic factor for GIST patients. According to a study published in 2018, of the nine genes screened by CRISPR-Cas9 technology that were most likely to lead to resistance against imatinib, SOCS6 was one of the most promising targets [22]. In the future, we will explore the associations between SOCS6 expression and proliferation and drug-resistance of GIST by performing in-vivo and in-vitro assays. Additionally, the mechanisms through which SOCS6 regulates proliferation and resistance against imatinib of GIST cells will also be investigated. However, some shortcomings of the present study are not totally to be neglected. Firstly, this study is a retrospective one in nature, meaning that selection bias is not absolutely avoidable. Secondly, the number of included patients is relatively small, warranting larger-scaled studies. Thirdly, the specific mechanisms have not been studied, suggesting further studies are needed to elucidate these mechanisms. Despite these drawbacks, the present study could still provide some valuable suggestions for future clinical practice and research given the fact that this is one of the few studies reporting the prognostic significance of SOCS6 in GIST.

## Conclusions

Low SOCS6 expression is an independent predictive factor for worse survival of GIST patients, suggesting its potential as a novel prognostic biomarker for GIST patients.

## Data Availability

The data analyzed in the present study are available from the corresponding author on reasonable requests.

## Abbreviations

SOCS6: suppressor of cytokine signaling 6
GIST: gastrointestinal stromal tumor
OS: overall survival
RFS: recurrence-free survival
PDGFRA: platelet-derived growth factor receptor-α
RPTK: receptor protein tyrosine kinase
LOF: loss of function
NIH: National Institutes of Health
TKI: tyrosine kinase inhibitor
BSA: bovine serum albumin
DAB: 3,3,-diaminobenzidine
HR: hazard ratio
95%CI: 95% confidential interval
HPF: high-power field
SH2: Src homologous domain
